# A micro-LED array based platform for spatio-temporal optogenetic control of various cardiac models

**DOI:** 10.1038/s41598-023-46149-1

**Published:** 2023-11-09

**Authors:** Sebastian Junge, Maria Elena Ricci Signorini, Masa Al Masri, Jan Gülink, Heiko Brüning, Leon Kasperek, Monika Szepes, Mine Bakar, Ina Gruh, Alexander Heisterkamp, Maria Leilani Torres-Mapa

**Affiliations:** 1 Institute of Quantum Optics, Gottfried Wilhelm Leibniz University, 30167 Hannover, Germany; 2 QubeDot GmbH, Wilhelmsgarten 3, 38100 Brunswick, Germany; 3 Department of Cardiac, Thoracic-, Transplantation and Vascular Surgery, Leibniz Research Laboratories for Biotechnology and Artificial Organs (LEBAO), Hannover Medical School, 30625 Hannover, Germany; 4 Lower Saxony Centre for Biomedical Engineering, Implant Research and Development (NIFE), 30625 Hannover, Germany

**Keywords:** Biophotonics, Cellular imaging, Biomedical engineering

## Abstract

Optogenetics relies on dynamic spatial and temporal control of light to address emerging fundamental and therapeutic questions in cardiac research. In this work, a compact micro-LED array, consisting of 16 × 16 pixels, is incorporated in a widefield fluorescence microscope for controlled light stimulation. We describe the optical design of the system that allows the micro-LED array to fully cover the field of view regardless of the imaging objective used. Various multicellular cardiac models are used in the experiments such as channelrhodopsin-2 expressing aggregates of cardiomyocytes, termed cardiac bodies, and bioartificial cardiac tissues derived from human induced pluripotent stem cells. The pacing efficiencies of the cardiac bodies and bioartificial cardiac tissues were characterized as a function of illumination time, number of switched-on pixels and frequency of stimulation. To demonstrate dynamic stimulation, steering of calcium waves in HL-1 cell monolayer expressing channelrhodopsin-2 was performed by applying different configurations of patterned light. This work shows that micro-LED arrays are powerful light sources for optogenetic control of contraction and calcium waves in cardiac monolayers, multicellular bodies as well as three-dimensional artificial cardiac tissues.

## Introduction

The discovery and cloning of channelrhodopsin-2 (ChR2)^1^ have paved the way for light-based control of membrane potential in mammalian cells. This approach is called optogenetics^2^ and has since then become an indispensable method to modulate and control cellular activity, intracellular signaling pathways as well as mapping neuronal activity^3^. Cell-specific opsin expression combined with the high temporal and spatial control enabled by light have made optogenetics a powerful tool for understanding and potential therapy of certain diseases such as Parkinson’s disease^4^ and retinitis pigmentosa^5^, as well as a promising future alternative for electrical cochlear implants^6^. In cardiac research, optogenetics was used to pace hearts of transgenic mice^7–10^, gain insights into the embryonic development of zebrafish hearts^11^ and explore non-excitable cells as potential pacemakers^12^. At present, there are multiple available genetically encoded actuators due to the continuous development of light-gated proteins with diverse kinetic properties, wavelength sensitivity and subcellular expression profile^13–16^.

As the optogenetic toolkit grows, so does the demand for versatile illumination sources and imaging techniques, both in vitro and in vivo. Especially the former is of high interest to enable precise delivery of the excitation light^17^. Commonly used light sources are lasers, because they can be focused and scanned across a sample. To achieve simultaneous stimulation at different locations in the sample, spatial light modulators (SLMs)^18–21^, digital mirror devices (DMDs)^9,10,22–24^ or acousto-optical modulation^8^ can be used to modulate the phase or amplitude of light and create the desired light patterns at the target plane. These devices can provide high spatial accuracy with refresh rates up to several kHz. However, implementing them in an optogenetic setup can require considerably high computational effort and costs. For this reason, easy-to-build and low-cost systems are in demand^25^.

Recent progress in gallium nitride (GaN) based micro-LED technology enabled production of devices with high pixel counts and variable pixel size. Self-emitting micro-LED arrays consisting of hundreds of pixels compressed on a small area provide inherent spatial resolution without additional beam shaping methods or expensive lasers. Furthermore, individual control of each pixel allows for projection of dynamic light pattern illumination on the samples^26^. Micro-LED arrays have been reported to optogenetically control neuronal activity in rats’ hippocampal neurons^27^, retinal ganglion neurons^28^ and in whole hippocampal slices^29^. Neural probes consisting of light emitting micro-LEDs or organic LEDs can be used to perform deep brain stimulation with high resolution and monitor cellular electrical responses simultaneously^30,31^. In combination with genetically encoded calcium indicators, micrometer-sized light emitters were used to trigger intracellular calcium transients in transgenic HEK 293 cells^32^. Most of these studies used the micro-LED for static illumination. However, dynamic light pattern illumination has yet to be demonstrated using these light sources. It was shown that the chirality of cardiac spiral waves and rotors, which play a crucial role in the formation of arrhythmia, can be reversed by dynamic blue light patterned illumination of counter-spiral patterns on ChR2 expressing cardiac cells^22^. For further research into the control of arrhythmia-inducing signal rotors—either to extinguish them or to create them—an easy-to-implement tool may be crucial for testing potential therapies^33,34^.

For the investigation of light-induced behavior in mammalian cells, human induced pluripotent stem cells (hiPSCs) represent a valuable and powerful tool. Numerous protocols have already been established for the differentiation of hiPSC in different cell types, including neurons and cardiomyocytes^35,36^. This virtually unlimited source of the desired cell types can also be combined with the integration of transgenes encoding for relevant proteins such as calcium indicators or light-sensitive channels under control of tissue specific or ubiquitous promoters. Recent papers have shown the progressive growing interest in the scientific community in using stem cell technology and optogenetics for developing a sophisticated biological model for investigating tissue electrophysiology and complex cellular interactions in physiologically relevant contexts, in a rapid and non-invasive manner, in particular in the cardiac field^37,38^.

In this work, we present a platform using a 16 × 16 directly-addressable micro-LED array which is incorporated in a fluorescence microscope and projected onto the sample plane. The chosen optics in our set-up ensure that the entire array of micro-LEDs is always scaled with the microscope’s magnification and thus, the field of view (FOV) is always fully covered by micro-LEDs. Using this platform, we demonstrate that a micro-LED array is capable of pacing light-sensitive cardiac bodies and functional bioartificial cardiac tissue produced from human induced pluripotent stem cell derived cardiomyocytes (hiPSC-CMs). Illumination of light patterns enables steering of calcium waves in ChR2 expressing cardiac cell monolayer. Calcium waves can be redirected by applying a temporally dynamic pattern of light with a user-defined arbitrary configuration.

Overall, this work demonstrates that self-emitting micro-LED arrays are flexible tools for both static and dynamic optogenetic control of cardiac activity.

## Materials and methods

Three different cardiac models were used in our study. Multicellular aggregates of highly purified cardiomyocytes, termed cardiac bodies^39^, and bioartificial cardiac tissue (BCT), a composition of cardiomyocytes and fibroblasts derived from human pluripotent stem cells (hiPSCs)^40^. A transgenic HL-1 cell line^41^ was used as a model for a 2D syncytium. All samples expressed the channelrhodopsin-2 variant H134R fused to eYFP. The hiPSCs were generated in house. The HL-1 cell line was kindly provided by Prof. Dr. Philipp Sasse. No animals were involved in this study.

### Human induced pluripotent stem cell line culture

The human pluripotent stem cell line MHHi009-A-3 constitutively expresses the channelrhodopsin gene (ChR2, H134R variant5) in fusion to an eYFP reporter gene under the control of the ubiquitous CAG promoter (AAVS1-pCAG-ChR2-eYFP) and Zeocin resistance gene (ZeoR) under the control of the cardiac-specific α-myosin heavy chain (pMYH6) promoter. The hiPSC monolayers were cultivated in E8 medium^42^ on Geltrex-coated flasks and passaged every 3–4 days using Accutase (both Life Technologies)^43^. Cells were kept at 37 °C and 5% CO_2_.

### Cardiac differentiation

**Figure 1:**
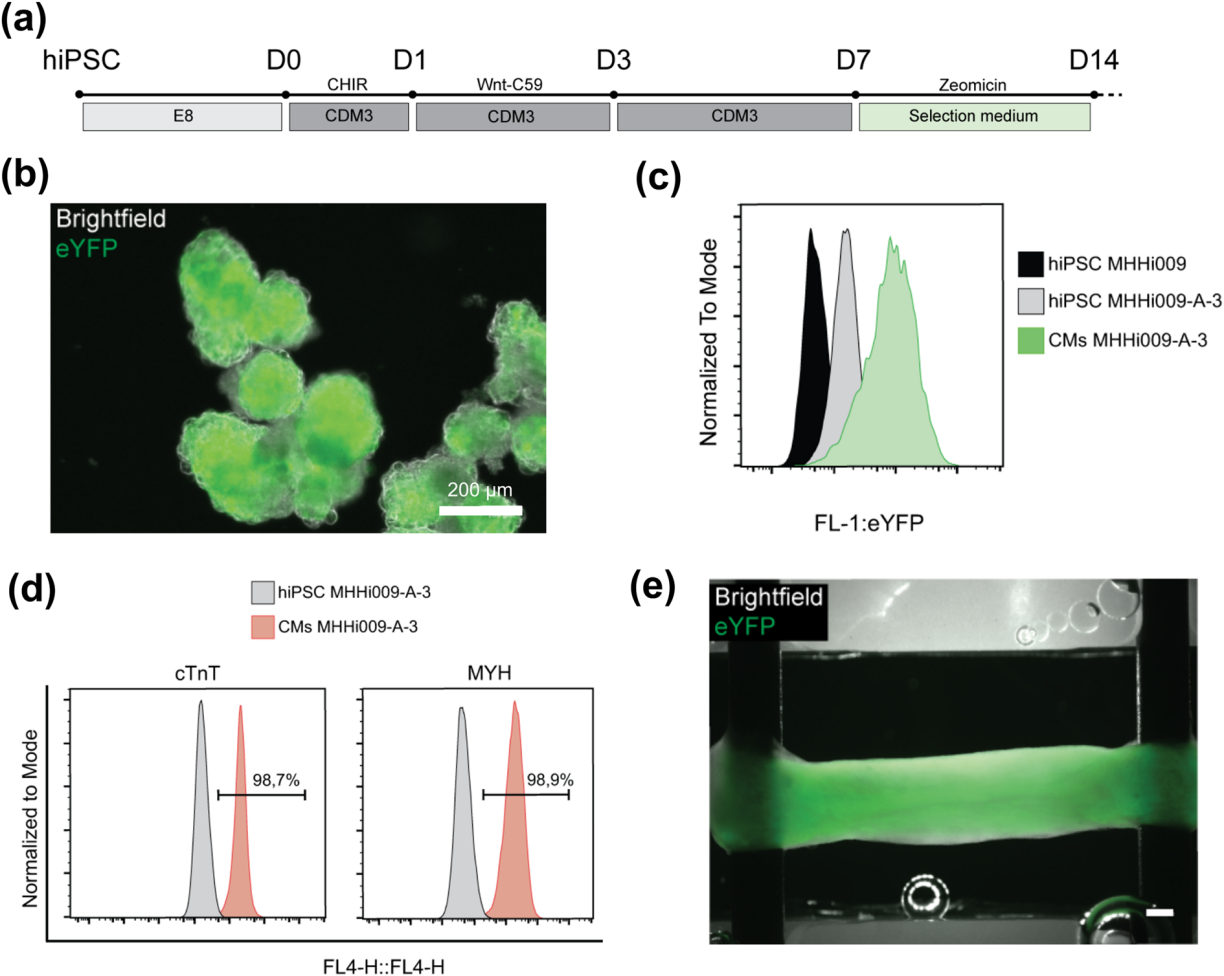
(**a**) Overview of the cardiac differentiation of MHHi009-A-3 through biphasic modulation of WNT pathway, followed by antibiotic selection using Zeomicin resistance gene (ZeoR). (**b**) Differentiation at D15 resulted in cardiac bodies constitutively expressing eYFP. (**c**) Flow cytometry analysis showed homogenous expression of eYFP in MHHi009-A-3 hiPSC and CMs, compared to the not transgenic mother stem cell line MHHi009-A. (**d**) Cardiac selection was performed adding Zeomicin to the culture medium for 7 days, resulting in high expression of cardiac markers cardiac troponin T (cTnT) and myosin heavy chain (MYH) at D15. (**e**) Bioartificial cardiac tissues were generated, combining cardiomyocytes with irradiated fibroblasts and hydrogel solution. At D21, progressive growing stretch and spontaneous remodeling of the matrix resulted in compacted, homogenous, and beating tissues. Scale bar is 500 μm.

A schematic outline of the cardiac differentiation protocol is shown in Fig. 1a. HiPSC were differentiated into cardiomyocytes (CMs) using the established protocol based on the biphasic modulation of the WNT pathway^39^. Briefly, hiPSC monolayers were dissociated in single cells using Accutase (D3), then seeded at 0.2 × 10^6^ cell/ml density in 125 ml Erlenmeyer flasks in E8 medium supplemented with 10 µM Rock Inhibitor (RI). Cells were kept in suspension culture on an orbital shaker (at 70 rpm at 37 °C and 5% CO2) for three days, allowing generation of 3D aggregates called embryoid bodies (EB). Differentiation was started with activation of the Wnt pathway using 5 µM CHIR99021 (Leibniz University, Hannover) for 24 h (D0), followed by the inhibition of Wnt pathway using 2 µM Wnt-C59 (Leibniz University, Hannover) for 72 h (D1–D3). Between D3 and D7, cells were cultivated in CDM-3 medium without any supplements and medium change was performed every other day.

Spontaneous contractions in the aggregates could be observed around D7. Fluorescence image of the cardiac bodies depicts the YFP signal from the ChR2 positive cells (Fig. 1b). Flow cytometry data shows high eYFP expression in transgenic hIPSC and differentiated cardiac cells MHHi009-A-3 compared to the mother cell lines MHHi009-A (Fig. 1c). At D8, cardiac selection was performed by using basic-serum-free medium (21969-DMEM, 1% Non-essential Amino Acids, 2 mM l-glutamine, 0.1 mM β-mercaptoethanol all from Life Technologies, 5.6 mg/l transferrin, 37.2 µg/l Sodium-selenite both from Sigma-Aldrich) supplemented with 100 µg/ml Zeomicin (Life Technologies) for 7 days. Efficiency of the cardiac differentiation and selection was analyzed via flow cytometry using typical cardiac markers, cardiac troponin T (cTnT) and myosin heavy chain (MYH) (96.7% and 98.9%, respectively) at day 15 (Fig. 1d).

### Flow cytometry

Immunostaining and flow cytometry analysis on single cell suspension were performed as previously described^43^. Prior to antibody staining for intracellular markers, cells were fixed with ice-cold 90% methanol (15 min). Primary antibodies or the respective isotype controls, diluted in a PBS-based buffer (0.1% Triton-X 100, 0.5% BSA; both Sigma-Aldrich) were incubated with the cells for 1 h, followed by a 30 min incubation period with the appropriate secondary antibody (Donkey Anti-mouse IgG Alexa Fluor 647; 1:300, from Dianova, DE). All measurements were performed with the Accuri C6 (BD Biosciences) flow cytometer and data was analyzed using FlowJo_V10 (FlowJo LLC). Primary antibodies and their dilutions are listed in Table 1.

**Table 1:** List of primary antibodies and their respective dilutions used for flow cytometry.

Antibody	Isotype	Company	Dilution
MYH1E (MF20) #AB_2147781	Mouse IgG2b	Hybridoma Bank, University of Iowa, US	1:50
Troponin-T #MS-295-P	Mouse IgG1	Richard Allan Scientific, Kalamazoo, US	1:100

Isotype controls			
Negative control	Mouse IgG2b	Dako, Glostrup, DK	
Negative control	Mouse IgG1	Dako, Glostrup, DK	

Isotype controls were used in the same concentration as the respective primary antibody for each staining.

### Bioartificial cardiac tissue

Cardiac bodies were dissociated in single cell suspension using STEMdiff™ Cardiomyocyte Dissociation Kit (Stem Cell Technologies) according to manufacturer’s instructions. Cardiomyocytes (1 × 10^6^ cells per tissue) were then combined with irradiated foreskin fibroblasts (1 × 10^5^ cells per tissue) in ratio of 10:1 into a hydrogel mixture composed of 0.9 mg/ml rat collagen type I (R&D System), 10% Geltrex™, and 2.5% 0.4 M NaOH (Sigma-Aldrich)^40^. The cell–matrix solution was poured into custom-made silicone molds with anchoring titanium rods at 6 mm distance and left for 30 min at 37 °C to allow solidification^44^. Bioartificial cardiac tissues (BCTs) were cultivated in BCT medium (DMEM F12 supplemented with 12% (v/v) horse serum (Life Technologies), 1 mM l-glutamine, 10 µg/ml insulin (Sigma-Aldrich), 1% penicillin–streptomycin (Life Technologies). Daily medium change was performed with fresh BCT medium supplemented with 30 µM l-ascorbic acid (Sigma-Aldrich). BCTs were cultivated for 7 days, then a progressive growing stretch was applied by stepwise distancing of the rods by 400 µm every 4 days until day 21^40, 44^. Hydrogels, such as the mixture of Geltrex and Collagen I, naturally stimulate cells to form intercellular connections, which reduces the liquid content and thereby remodels the cardiac tissue^44^. This was observed for the BCTs used in this study, too, by monitoring the reorganization of the tissue using stereomicroscopy pictures over the 21 days of culture. A reduction in tissue diameter of approximately 40% was observed. Increased cellular interconnectivity was also demonstrated through the transition from sporadic contractions in the early days after production to a more synchronized and uniform contraction by day 21.

Figure 1e shows the fluorescence image of a bioartificial cardiac tissue with a uniform YFP expression on the entire tissue.

### HL-1 cell line

As a 2D cardiac syncytium model, we used a stably ChR2(H134R)-expressing HL-1 cell line reported in the following studies^18, 45–47^. The HL-1 cells used for this study were genetically modified via lentiviral transduction of EF1α-ChR(H134R)-EYFP.

Successfully transduced cells were isolated via fluorescence assisted cell sorting and maintained in Claycomb medium (Sigma-Aldrich) supplemented with 10% fetal bovine serum (Sigma-Aldrich), 1% l-glutamine (Sigma-Aldrich), 1% norepinephrine (Sigma-Aldrich), and 1% penicillin and streptomycin (P/S, Biochrome). The medium was refreshed each day and cells used for these experiments were kept at low passage numbers (p < 30).

The sample preparation and staining were discussed in our previous study^18^. Briefly, 100,000 HL-1 cells expressing ChR2 were seeded in a 35 mm glass bottom dish (ibidi) coated with fibronectin (5 mg/l, Sigma-Aldrich) and gelatin (0.02%, Sigma-Aldrich) and incubated for further 24 to 48 h prior experiments. On the day of the experiments, cells were washed with Dulbecco phosphate buffer solution (DPBS, PanBiotech) without Ca^2+^ and Mg^2+^ and stained with 2 µl of 5 mM Cal-630 in 500 µl culture medium. After 45 min of incubation, the medium was replaced with 1 ml of fresh medium and cells were further incubated for 30–45 min before optogenetic experiments.

## Experimental setup

### Imaging

**Figure 2:**
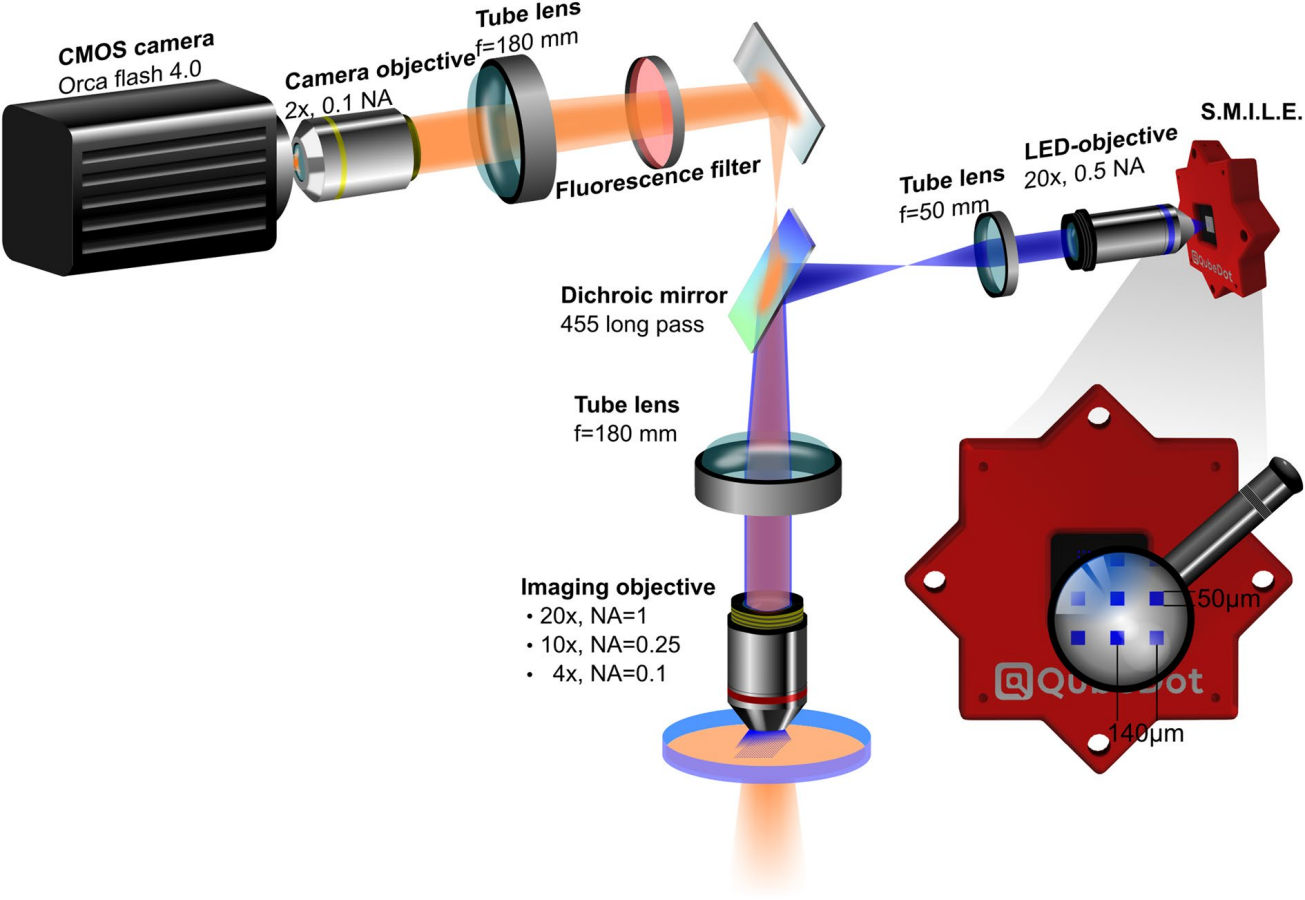
Imaging platform with integrated SMILE. Samples were illuminated from below and imaged from above by a 20×, 10×, or 4× objective with matching tube lens (f = 180 mm). Another pair of objective and tube lens demagnifies the image by 1.8 and projects it onto the CMOS chip. Blue light with a central wavelength of 450 nm emitted SMILE is captured by a 20× objective in combination with a tube lens (f = 50 mm) and guided onto the sample by a dichroic mirror (455 nm long pass). Tube lenses are adjusted in 4f-arrangement.

A schematic diagram of the setup is shown in Fig. 2. The custom designed microscope consists of a pair of objectives, one in front of the camera (2×, 0.1 NA, Nikon, camera objective) and a second one directly above the sample (4×, 0.1 NA/10×, 0.25 NA/20×, 1 NA, Olympus, imaging objective). The objectives work with a corresponding tube lens (AC508-180-AB-ML, f = 180 mm, Thorlabs) arranged in a 4f configuration, as depicted in Fig. 2, to first magnify and then demagnify the image of the sample on the CMOS camera chip (Orca flash 4.0, Hamamatsu). The size of the field of view can be flexibly changed by using different imaging objectives.

Video microscopy of the contraction of the cardiac bodies and BCTs were performed in transillumination mode by an LED with a central wavelength of 590 nm (Solis-590C, Thorlabs).

For fluorescence imaging, an excitation filter (605/15, FF01-605/15-25, Semrock) was used to narrow the LED spectrum and an emission filter (655/40, FF02-655/40-25, Semrock) was integrated between the two tube lenses. The light source and filter set were chosen to match the excitation and emission spectrum of the fluorescent calcium dye, Cal-630.

### Micro-LED array

Optical excitation of ChR2-expressing cells was realized using a Structured Micro Illumination Light Engine (SMILE, QubeDot) consisting of 256 squared pixels of micrometer-sized light emitting semiconductors arranged in an array of 16 × 16. Each single micro-LED is 50 × 50 µm^2^ in size with a pixel pitch of 140 µm. SMILE was powered and controlled via a 5 V USB-cable connected to a computer. Each single pixel could be switched on and off independently by the provided software, which was also used to create looped animations. The emitted blue light (450 nm, central wavelength) was collected by an objective (20×, 0.5 NA, dry, Nikon) and a tube lens (f = 50 mm, Thorlabs) and implemented into the microscope by a dichroic mirror (T455 LPXR, Semrock) in between the 180 mm tube lenses, as depicted in Fig. 2.

### Experimental methods

The emission power of the micro-LED array was measured via an optical power meter (PWM100USB, Thorlabs) either directly in front of the device to quantify the direct light output or at the sample plane to measure the power reaching the sample.

Periodic light excitation of the cardiac bodies was realized by creating animations consisting of single frames in the SMILE software. Depending on the desired illumination time and pacing frequency, one frame with active pixels was followed by a respective amount of empty frames. The refresh rate, which corresponds to the amount of time each frame was active, was dictated by the number of empty frames. Each animation was prepared in advance and stored as graphics interchange format (GIF).

All videos were captured via a custom-made LabView program stored in tagged image file format (tiff) and binned to reduce the file size (for brightfield configuration) or to increase the signal-to-noise ratio (for fluorescence mode). Analysis of amplitude and duration of contraction was conducted by Myocyter^48^. The relatively large contraction amplitude rendered any motion artifacts neglectable, for example floating tissue residuals in the medium. In some samples, light from the micro-LEDs was visible in the camera, which can impede the image analysis. Therefore, a region of interest (ROI) was chosen to contain the outlines of the respective CB.

For optogenetic experiments, the cardiac bodies were placed in 35 mm dishes (ibidi) in complete medium and incubated at 37 °C and 5% CO_2_. After 1–3 days, they would attach to the bottom of the dish and were imaged using the 10× imaging objective. BCTs were incubated at 37 °C and 5% CO_2_. For imaging they were transferred to a 35 mm glass bottom dish with one of the titanium rods carefully removed to facilitate visible contraction. The BCTs were imaged with the 4× imaging objective. HL-1 cells expressing ChR2 were prepared and stained with Cal-630 dye as mentioned above. Calcium imaging was performed using the 20× imaging objective.

To acquire activation maps for steering of the calcium waves in 2D HL-1 syncytia, the Matlab-toolbox COSMAS^49^ with a self-written GUI was used after applying a spatial Gaussian filter with 10 pixel radius in Fiji to the tiff images. For the wave steering experiments implemented in this work, calcium waves prior to and after the applied patterns were analyzed and plotted as activation maps.

All experiments on biological samples were carried out within limited time periods (less than 30 min) at room temperature (20 °C).

## Experimental results

### Characterization of imaging setup

In our system, the sample plane is imaged onto the CMOS chip by two compound microscopes, a magnifying one in the forward and a demagnifying one in the backward direction. Since the magnification is only valid for the focal length of the manufacturer’s specified tube lens, the effective magnification of an objective (*M*_*Effective*_) is given by

1 $$M_{Effective} = M_{Design} \frac{{f_{Microscope} }}{{f_{Design} }},$$

where *M*_*Design*_ represents the denoted objective magnification, *f*_*Microscope*_ the tube lens’ focal length used in this work and *f*_*Design*_ is the focal length of the tube lens as specified by the manufacturer. To magnify each sample, a 4×, 10× or 20× objective was used with a matching tube lens (f = 180 mm). A demagnifying pair of a 2× objective (Nikon, *f*_*Design*_ = 200 mm) and a 180 mm tube lens yields *M*_*Effective*_ = 1.8×. This leads to a total magnification of 2.2×, 5.6× or 11.1× of the sample at the camera chip. The corresponding field of view was measured via Fiji to be 3 mm, 1.1 mm and 0.55 mm, respectively.

**Figure 3:**
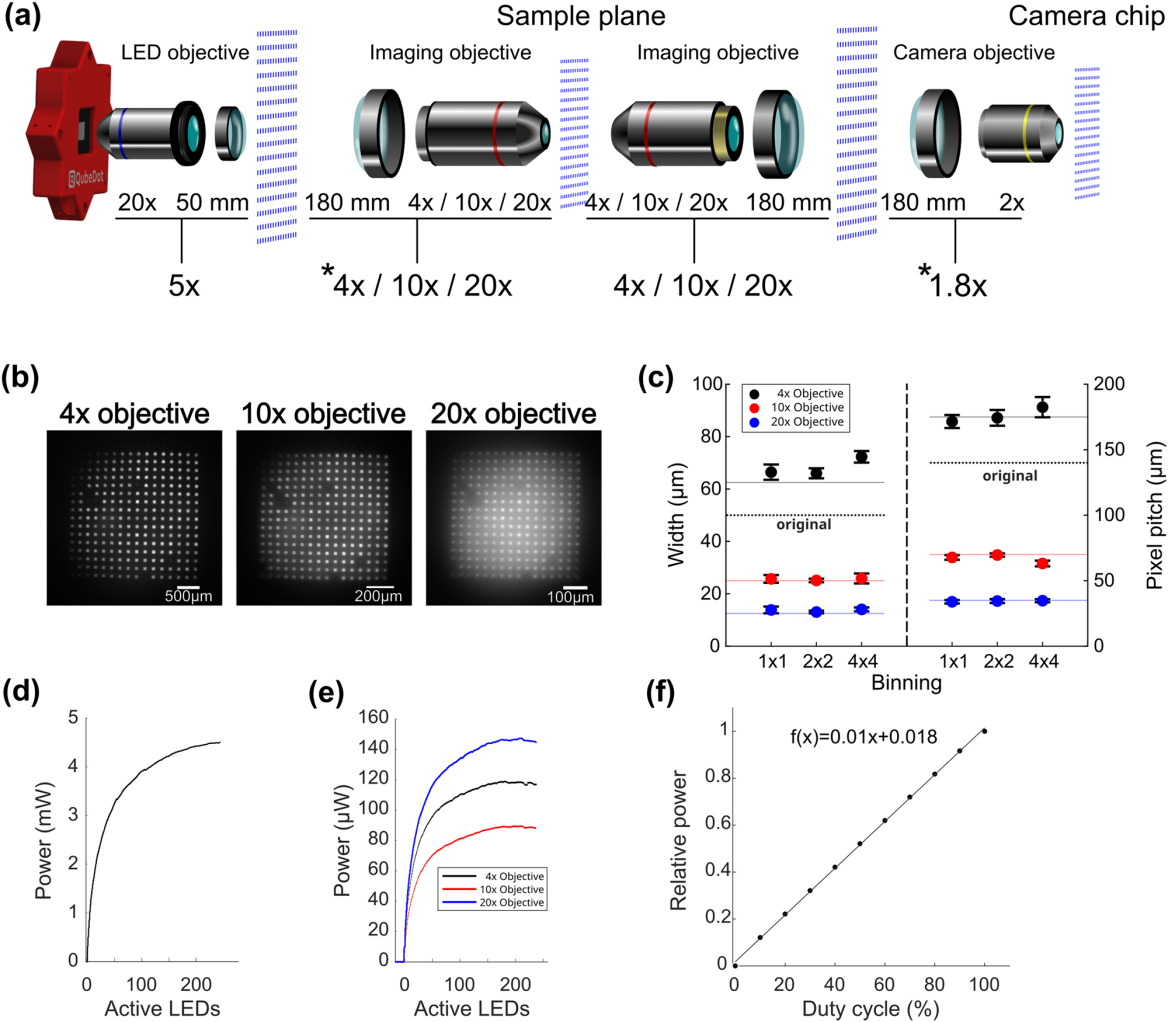
(**a**) Sketch of the light’s imaging path emitted by the SMILE. Each depicted array of blue micro-LEDs is in the focal length of the respective tube lens or objective. Below each objective, the denoted magnification and used tube lens’ focal length are given, as well as the actual magnification. Depicted micro-LED arrays are not to scale. Asterisks denote demagnification by the respective segment. (**b**) Images of the whole array at the sample plane for 4×, 10× and 20× imaging objectives. (**c**) Plot of pixel size and pitch for the three imaging objectives with corresponding camera binning factors 1, 2 and 4. Dots represent mean values of 15 measured pixels with corresponding standard deviation. Pale lines depict the theoretical values according to Table 2. Bold dashed lines show original pixel width and pitch. (**d**) Power output of the micro-LED array as a function of switched on pixels close to the device and (**e**) at the sample plane. (**f**) Relative power output of the micro-LED array in duty cycles from 0 to 100%, measured at the sample plane.

To magnify the micro-LED array, a 20× objective (Nikon) with an f = 50 mm tube lens was used, which reduced the designed magnification from *M*_*Design*_ = 20× (*f*_*Design*_ = 200 mm) to *M*_*Effective*_ = 4×. In combination with the imaging objectives, the micro-LED array was imaged onto the sample with a scaling factor of either 1.25, 0.5 or 0.25. The beam path of the imaged micro-LEDs is depicted in Fig. 3a. Since it passes through the imaging objective, the size of the imaged micro-LED scales with the magnification of the microscope itself. Residual blue light from the sample plane, imaged onto the camera despite the dichroic mirror, always irradiates the same area at the CMOS chip as shown in Fig. 3b.

The sizes of a single pixel at the sample plane can be calculated as the product of the magnification of both imaging ensembles, which in theory are 62.5 µm, 25 µm or 12.5 µm for the three objectives, respectively. Figure 3c shows the experimentally measured pixel size and pitch as a function of camera binning. Due to the lower spatial resolution, the pixels and their pitch size can appear slightly bigger or smaller at the camera chip than the theoretical value (Fig. 3c pale lines) when using small magnification or higher binning.

Each single pixel in the micro-LED array provides at least 300 µW of output power, but does not scale linearly with increasing number of switched-on pixels. This is attributed to the common ground contact (cathode) and its resistance of the LED array. A higher amount of switched-on pixels leads to a higher current, which leads to a higher voltage loss at the common ground contact for all pixels and therefore reducing the current and optical output power of individual micro-LEDs. This behavior is called “multi pixel droop”^50^. A maximum power of 4.5 mW can be reached, when all pixels are switched on at the same time (Fig. 3d). The light reaching the sample plane is of significantly lower power: 120 µW, 90 µW or 149 µW maximum for the 4×, 10× and 20× imaging objective, respectively (Fig. 3e). The corresponding intensities at the sample plane are inversely related to the numbers of used pixels due to the multi pixel droop. Therefore, the maximum intensities of 1.7 mW/mm^2^, 8.5 mW/mm^2^ and 52.3 mW/mm^2^ for the 4×, 10× and 20× objectives, respectively, are achieved when only a single micro-LED is used. Precise control of the applied power at the sample plane can be achieved by pulse width modulation (PWM). It can be operated at a user-defined duty cycle with a minimum of 130 µs frame-time for the used device. For example, an animation consisting of one active and one inactive frame, looped in a frame rate yields an average power reduction of 50%. Here, PWM was tested and verified for 200 µs illumination time per frame. The power output is linearly proportional to the duty cycle (Fig. 3f). Table 2 summarizes the specifications of the setup using the different imaging objectives used.

**Table 2:** Specifications of micro-LED array and microscope, depending on the respective imaging objective.

	Product name	UPlanFL N	MPlanFL N	XLUMPlanFL N
Objectives	Magnification	4×	10×	20×
	Numerical aperture	0.1	0.3	1
	Immersion	Dry	Dry	Water
Microscope	Total magnification of the sample	2.2×	5.6×	11.1×
	Field of View (diameter)	3 mm	1.1 mm	0.55 mm
Micro-LEDs	Magnification of micro-LED array at sample plane	1.25×	0.5×	0.25×
	Pixel width at sample plane	62.5 µm	25 µm	12.5 µm
	Pixel pitch at sample plane	175 µm	70 µm	35 µm
	Maximum power at sample plane	120 µW	90 µW	149 µW

All the used objectives were manufactured by Evident (formerly Olympus).

### Optical pacing of light-sensitive hiPSC cardiac bodies and bioartificial cardiac tissues

**Figure 4:**
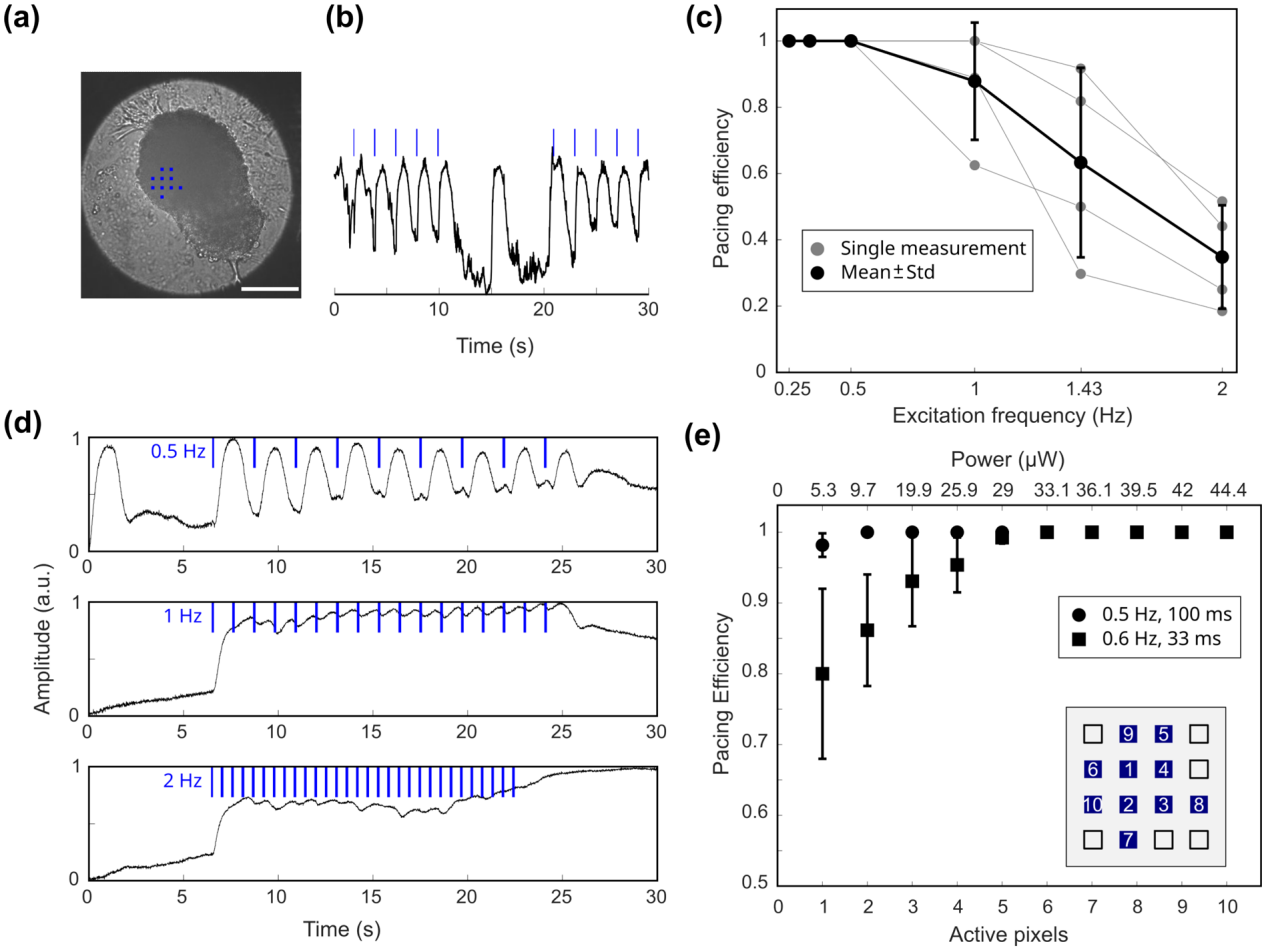
Optogenetic excitation of cardiac bodies. (**a**) Image of a cardiac body stimulated with indicated pattern of micro-LEDs (blue dots, scale bar: 200 µm) and (**b**) a contraction trace of 5 paces at 0.5 Hz with a 10 s pause followed by other 5 illuminations. Light-triggered and spontaneous peaks in contraction are clearly visible. (**c**) Dependence of pacing efficiency of cardiac bodies on pacing frequency (0.25 Hz—2 Hz, n = 4, 10 pixels). Black line represents mean values ± standard deviation. (**d**) Representative contraction traces for pacing frequencies of 0.5 Hz, 1 Hz and 2 Hz with 100 ms illumination time and 30 µW (10 pixels). Contraction becomes irregular at 2 Hz. (**e**) Pacing efficiency as a function of number of active pixels for 0.5 Hz and 100 ms illumination time (circles, n = 24) and 0.6 Hz and 33 ms illumination time (squares, n = 27). Markers represent mean values ± standard deviation. The order of switched on pixels is given in the inset.

Cardiac bodies were examined using the 10× imaging objective in bright field mode (Fig. 4a). Illumination with blue light triggered contractions which were distinct from spontaneous contractions (Fig. 4b). Pacing the clusters was carried out at different pacing frequencies from 0.25 to 2 Hz using an illumination time of τ = 100 ms. We defined pacing efficiency as the relative number of successfully light-triggered contractions. While slower pacing frequencies yielded 100%, frequencies of 1 Hz and higher did not always elicit 1:1 contraction (Fig. 4c). Figure 4d shows examples of amplitudes for one cardiac body. While 0.5 Hz and 1 Hz pacing result in peaks clearly correlated to the excitatory stimulus, 2 Hz pacing led to irregular and unpredictable contractions.

We observe that most of the time, a single switched-on pixel with 5.3 µW at the sample plane is sufficient to successfully trigger a contraction at 0.5 Hz and τ = 100 ms. For shorter illumination time, τ = 33 ms and slightly higher pacing frequency, f = 0.6 Hz, the pacing efficiency decreased to 80% when illuminated with a single pixel, whereas six pixels, with total power of 21.7 µW at the sample plane were sufficient to reach a pacing efficiency of 100% (Fig. 4e, squares, n = 27).

**Figure 5:**
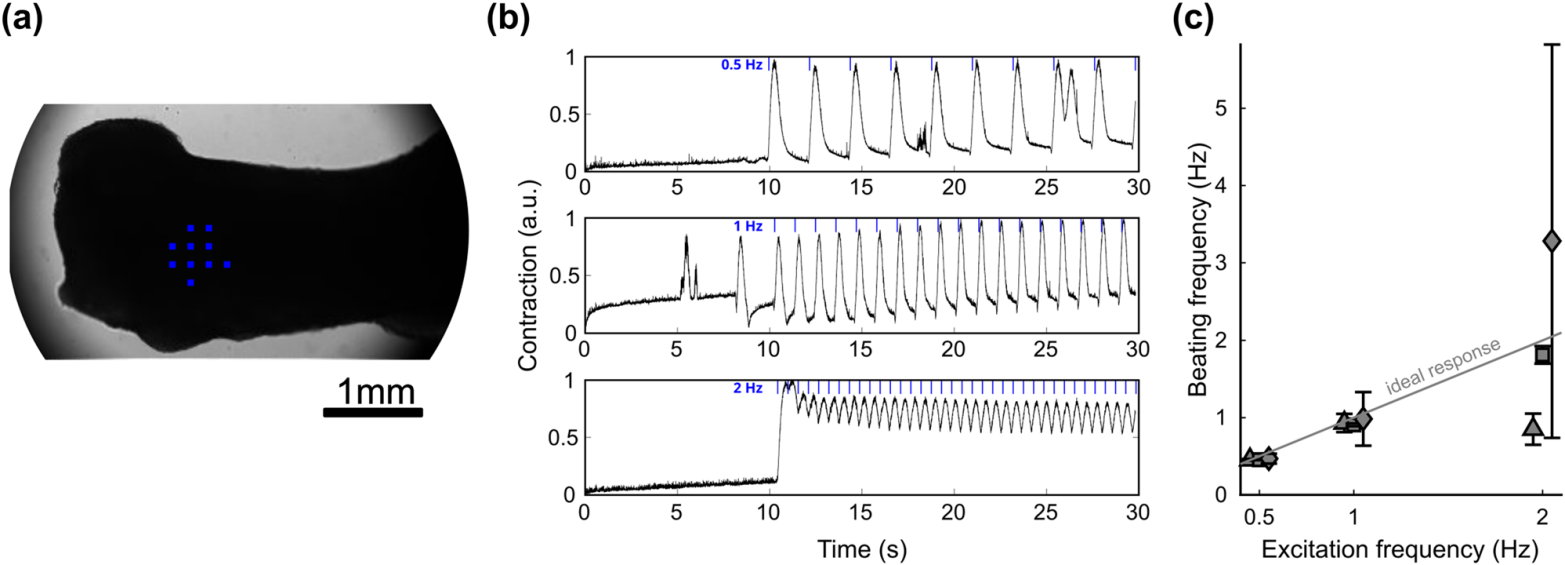
(**a**) Image of a cardiac body stimulated with indicated position of micro-LEDs by blue dots. (**b**) Sample traces of one BCT’s contraction at different pacing frequencies. (**c**) Frequency of contraction dependent on excitation frequency at 36 µW illumination power. Each symbol represents one BCT sample. While one sample (square) nearly exhibited 1:1 pacing all the time, the others followed the pacing at 0.5 and 1 Hz but contracted at half the pacing frequency (triangle) or completely irregular (diamond) at 2 Hz excitation. Error bars indicate standard deviation.

For the BCTs which were ~ 1 mm in width and over 5 mm in length (Fig. 5a), the 4× imaging objective was used. BCTs were paced with 10 micro-LEDs at frequencies of 0.5 Hz, 1 Hz and 2 Hz (Fig. 5b). Three BCTs were investigated. One of the BCTs followed the pacing frequency of the micro-LED array (Fig. 5c squares) for all frequency tested, while one was successfully paced at 0.5 Hz and 1 Hz, but skipped every second excitation trigger at 2 Hz (Fig. 5c triangles). The contractions of the third BCT (Fig. 5c diamonds) were highly unpredictable, especially at 2 Hz pacing frequency (Fig. S2). Contraction frequencies were calculated as the inverse of the time interval between the two peak maxima.

### Optical wave steering in a 2D cardiac syncytium

**Figure 6:**
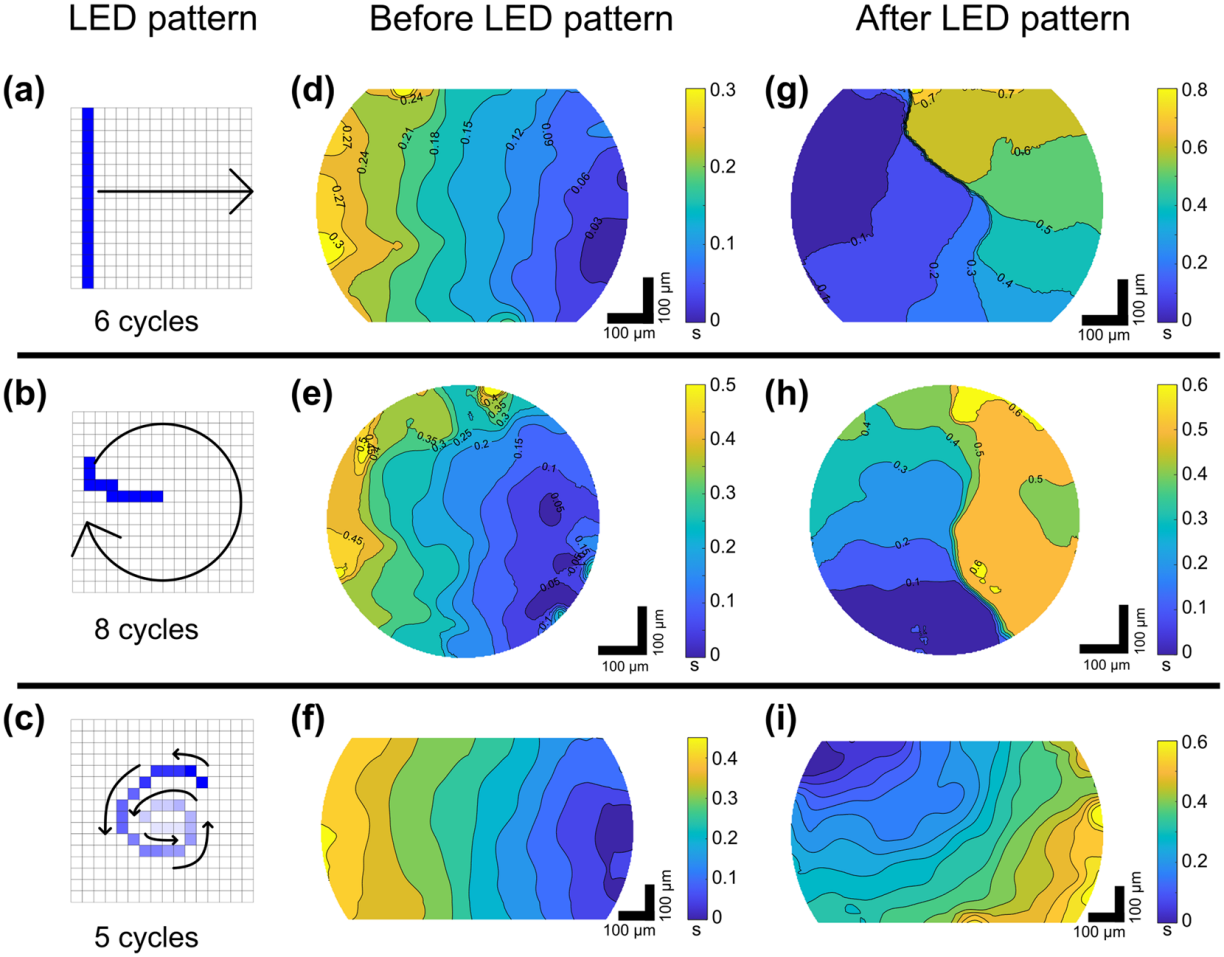
Representative activation maps of steered cardiac waves in HL-1 cells for three different animation patterns. (**a**–**c**) are the applied light patterns. (**d**–**f**) are the activation maps before applying the light patterns, (**g**–**i**) are the corresponding activation maps, respectively. For each map, the scale and timescale have been individually adjusted to better highlight the activation times of every pattern.

The micro-LED array provides a straightforward and intuitive interface to control each single emitter. Beside customized patterns of illumination, dynamic patterns are of crucial interest in cardiac optogenetic research, for example to change the chirality of native behavior of action potentials in ChR2-expressing cardiac monolayers^22^. In this section, we demonstrate dynamic light patterning using the micro-LED array to control calcium activity in 2D light-sensitive HL-1 cardiac monolayer. HL-1 cells are known to exhibit spontaneous spiral rotors or re-entries^51^. Three different dynamic light patterns for controlling and redirecting calcium waves were applied onto 2D syncytia of HL-1 cells: (1) a linear swipe (6 cycles at 0.6 Hz repetition rate, Fig. 6a), (2) a curved swipe rotated in a spiral around the center of the FOV (8 cycles at 0.5 Hz repetition rate, Fig. 6b), and (3) an inward-directed spiral (5 cycles at 0.6 Hz repetition rate, Fig. 6c). Each dynamic pattern, or animation, was created in the SMILE software as a sequence of single static patterns. Since HL-1 cells exhibit random spontaneous waves which could vary from dish to dish, illumination patterns were applied sequentially at arbitrary time points during the experiment. Representative activation maps of calcium waves before and after application of micro-LEDs are shown in Fig. 6d–f, g–i, respectively. For all patterns, the calcium waves changed their direction and followed the general trajectory of the light pattern.

The linear swipe was applied six times before the LEDs were switched off again. Figure 6d shows the calcium wave propagating from right to left of the field of view. Applying a linear, vertical train of blue light to the sample reversed the calcium wave direction from its initial leftwards flow direction towards right (Fig. 6g). Even though conduction was unimpeded prior to micro-LED application, the stimulated wave did not propagate through the upper half of the FOV, leading to a curved or spiral trajectory.

The curved pattern was applied and rotated eight times around the center of FOV before the LEDs were switched off again. Directly after light application, the initial leftwards directed flow (Fig. 6e) was changed and a spiral pattern of calcium wave could be observed (Fig. 6h). However, the spontaneous calcium wave immediately dominated after a single stimulated spiral trajectory. Hence, in this example, only a temporary modulation of calcium waves was implemented.

The inward directed spiral light pattern led to a clear directed shift in calcium wave propagation. Instead of a spontaneous leftward flow (Fig. 6f), the stimulated calcium wave came from the top left and spread towards the bottom right (Fig. 6i), after the light pattern was applied five times. In contrast to the second pattern, this behavior was maintained for succeeding waves.

To quantify the success rates of the applied pattern, we rated a change of the initial directory of the calcium wave to be either successful (the wave followed the applied pattern), half successful (the calcium wave did not follow the applied pattern but drastically changed its direction), or not successful (the applied pattern had negligible influence on the calcium wave’s direction). With this set of criteria, the linear swipe yields a success rate of 30% (n = 5), the curved rotating swipe yields 50% (n = 3), and the inwards directed spiral yields 33% (n = 3). These results are listed in Table 3 together with a depiction of the initial and resulting calcium wave’s direction.

**Table 3:**
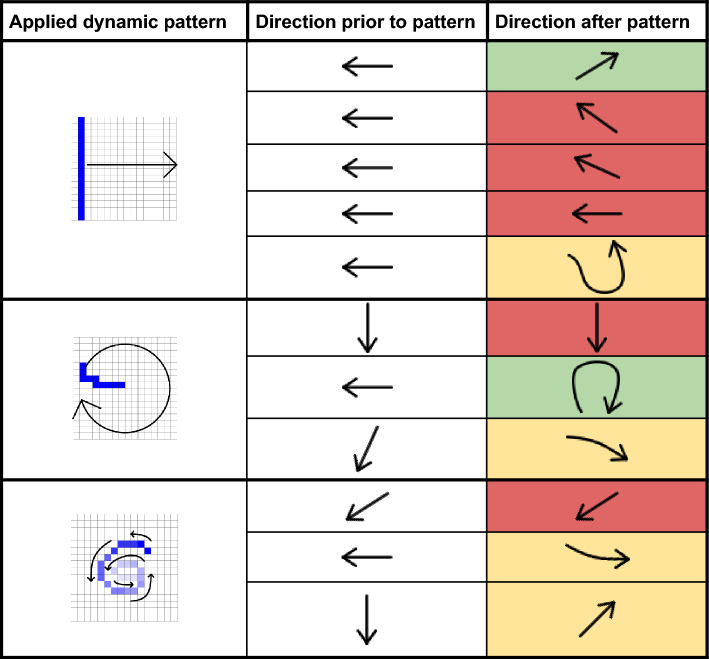
Direction of calcium waves in transgenic HL-1 cells prior to and after three different dynamic patterns of blue light excitation.

Green marked cells indicate calcium waves which followed the applied pattern, yellow ones were considered half successful and red ones non-successful.

## Discussion

We described a micro-LED platform for optogenetic actuation of ChR2-expressing cardiac models in vitro. The system is applicable for optogenetic control of a single cell, 2D monolayer up to whole tissue studies with the potential for high throughput data acquisition. The key component of our platform is the SMILE, a 16 × 16 array of 450 nm-emitting micro-LEDs which is imaged onto the respective sample. SMILEs can be produced with different emission wavelengths, with peak emission wavelengths ranging from 365 to 620 nm. Thus, the system is not only applicable to excite blue light sensitive ChR2, but could also be used to control other microbial opsins. For example, green emitting LED arrays could be used to inhibit cells expressing the outward proton pump, ArchT^52^. Whereas, red-emitting LED arrays would be useful for optogenetic stimulation of opsins which have optimal sensitivity at the orange to red wavelengths such as, Chrimson (λ ~ 590 nm^53^), ChRmine (λ 500–600 nm^54^) or ReaChR (λ ~ 590 nm and 630 nm^15^).

The magnification and demagnification of the micro-LEDs scale with the magnification and the FOV of our microscope itself, ensuring the entire FOV can be covered by blue light without the need to change or adjust the illumination source. The total FOV and magnification of the microscope can be easily matched to the sample of interest. Furthermore, for each magnification we triggered and imaged contractions of an optogenetic cardiac model with a corresponding dimension scale, thus confirming the potential of this set-up.

A single 50 × 50 µm^2^-sized pixel from the SMILE directly emits 300 µW, corresponding to an intensity of 120 mW/mm^2^—two orders of magnitudes higher than the necessary intensity to excite ChR2^55^. Although, the irradiance at the sample is considerably lower, ~ 1.7 mW/mm^2^, 8.5 mW/mm^2^ and 52.3 mW/mm^2^ for the 4×, 10× and 20× objectives, respectively, these intensities are still sufficient to pace the cardiac samples. Optical power losses in the system could be further reduced by incorporating a collecting lens directly attached to the micro-LEDs. An advantage of using SMILE is that each single pixel can be individually addressed without any cross-talk between neighboring pixels^56^. This maximizes the flexibility of our system for simultaneous and dynamic excitation.

In addition to its flexibility, our platform is easily customizable to targeted sample size, making it also suitable for research at the scale of whole tissue slices. The emergence of hiPSC-CMs and their potential as a tool to model arrhythmogenic diseases have been vastly discussed^57–59^ and non-invasive methods for control and analysis of such samples are demanded. We used multicellular cardiac bodies made of aggregated hiPSC-CMs as a model to show that our system is able to fulfill this demand. The intensity of a single switched-on pixel, 8.5 mW/mm^2^ using 10× objective is sufficient to trigger contractions in our cardiac bodies with 97% success rate at 0.5 Hz and 100 ms illumination time. Slightly higher frequency and lower illumination time of 0.6 Hz and 33 ms yielded a success rate of 80% ± 12%.

Another scope of application, we explored in our work, lies in the rising interest of engineered cardiac patches. Despite progress in cardiac research, cardiac diseases are still the main factor for adult mortality and morbidity. Therefore, regeneration of cardiac function is still an important ongoing research field^60, 61^. Cardiac tissue patches are currently being explored as candidates to aid repair and restore cardiac functionality. For example, hiPSC-CMs have been successfully used as implants in damaged hearts in animal models to improve cardiac function^62–65^. Recently, they have been proven to successfully integrate into the host myocardium of nonhuman primates^66^. However, electric coupling of the graft might be insufficient and thus a potential risk of arrhythmia, which is why modulation of the contraction frequency would be useful. The use of light-sensitive hiPSC-CMs and BCTs allows for thorough investigation in vitro and in vivo before considering clinical use of hiPSC-CMs. With our setup, we showed successful optical stimulation of cardiac patches, while parameters such as contraction frequency (Fig. 5c), amplitude (Fig. S1b), duration of contraction (Fig. S1c) and time to peak (Fig. S1d) and relaxation time (Fig. S1e), can be measured by all-optical means. These results display the potential of the setup for studies in cardiac patch therapy.

The micro-LED array can serve as an easy-to-use platform for high-throughput, contactless quantification of electrophysiological properties^67^. Such technology is of high interest in the context of cardiotoxicity screening for drug development^68^ or therapeutic applications e.g. treatment of short QT syndrome^69^. Termination of ventricular tachycardia has been demonstrated in ChR2-transgenic mouse hearts^8^ showing that termination success rate was highly dependent on the chosen illumination pattern. Feola et al.^70^ demonstrated that specific patterns of blue light applied on a monolayer of ChR2-expressing neonatal rat cardiomyocytes could successfully block reentrant spirals and also used a predefined illumination pattern protocol to create the initial reentrant calcium wave. These studies highlight the need for customized stimulation patterns for treatment of arrhythmogenic conditions.

As a final application, we demonstrate that our system can steer and redirect propagating waves of cardiac action potential in a 2D syncytium model cell line. By switching on and off successive lines of the micro-LED array, we created a dynamic pattern of light that can overwrite an existing spontaneous wave. By creating a spiral animation, we redirected an initially plane wave into a spiral one. This can also be performed by applying precisely timed static illumination, for example in an S1–S2 protocol^38^. Our approach does not rely on precise timing of optogenetic activation, but on repeated application of the desired patterns. However, since the pattern of light is applied to a small region of interest within the sample, the resulting calcium wave is highly influenced by the interaction of the stimulated wave and the irregular propagating spontaneous calcium waves, as well as to conduction blocks existing in the HL-1 cell monolayer. This is shown in Fig. 6a, d, g wherein a linear swipe results in a curved calcium wave trajectory, and in the relative low success rate for steering in general, which did not exceed 50%.

For future studies, it would be interesting to investigate the influence of the used patterns on the success rate of optical wave steering. Contrary to the other two patterns, spirally rotating waves of cardiac action potential are known to be stable because they are self-sustaining^70, 71^. To overcome the influence of calcium waves occurring outside the FOV, one can project the light patterns from the micro-LEDs on a larger region on the sample. In addition, several illumination parameters could be further optimized, such as the frequency, speed and timing of the applied dynamic light pattern.

Our experiments demonstrate the micro-LED array's high potential for investigating macroscopic waves in cardiac research. Implementation of the device has the potential to enhance and streamline current protocols for inducing and terminating spiral waves^72^. The ability to shape the excitation waves may be used for fast testing of anti-arrhythmic strategies, which might be even faster than computational models^73^. Thus, the simplicity of the micro-LED device is a promising approach for future investigations on optogenetic cardiac electrophysiology in vitro.

### Limitations of the study

The presented study has certain limitations for in vitro cardiac research. Particularly, the current system has a limited illuminated region in a large cell culture area. Any spontaneous electrical propagation originating outside of this region would have a huge influence on the illuminated region’s electrophysiology because they would interact with optogenetically steered electrical patterns. This scenario could be overcome by enlarging the illuminated FOV, which would require the use of a low magnification, high NA objective with significant light collection efficiency for fluorescence imaging. Furthermore, the presented optical system will benefit from an additional temperature control, which will be crucial for reliable and reproducible analysis.

At present, the micro-LED has a limited number of frames (2000) to build an animation. This constrains the use of PWM for pacing of cardiac models. In this case, an external shutter can be used to allow illumination with the desired frequency.

## Conclusion

We presented a microscope platform for optogenetic cardiac research with a plug-and-play micro-LED array consisting of 16 × 16 directly-addressable blue-emitting micrometer-sized pixels. We showed the usefulness of our system as a flexible and easy-to-use tool for cardiac optogenetics to analyze contractile behavior of hiPSC-CMs and bioartificial cardiac tissue. 10 micro-LEDs were shown to be sufficient for reliable cardiac pacing. Additionally, we illuminated dynamic light patterns using the inherent animation function of the SMILE to perturb and control the propagation of calcium waves in 2D syncytia of HL-1 cells. By careful design of our system, we ensured complete coverage of the FOV by the micro-LEDs independent of the chosen magnifying objective. The combination of high spatio-temporal resolution and parallel stimulation of the system can serve as an easy and powerful platform in cardiac optogenetic research.

### Supplementary Information

Supplementary Figures.

## Data Availability

The datasets used and analyzed during the current study are available from the corresponding author on request.
