# Author Correction: A micro-LED array based platform for spatio-temporal optogenetic control of various cardiac models

**DOI:** 10.1038/s41598-024-70488-2

**Published:** 2024-08-27

**Authors:** Sebastian Junge, Maria Elena Ricci Signorini, Masa Al Masri, Jan Gülink, Heiko Brüning, Leon Kasperek, Monika Szepes, Mine Bakar, Ina Gruh, Alexander Heisterkamp, Maria Leilani Torres‑Mapa

**Affiliations:** 1 Institute of Quantum Optics, Gottfried Wilhelm Leibniz University, 30167 Hannover, Germany; 2 QubeDot GmbH, Wilhelmsgarten 3, 38100 Brunswick, Germany; 3 Department of Cardiac, Thoracic‑, Transplantation and Vascular Surgery, Leibniz Research Laboratories for Biotechnology and Artificial Organs (LEBAO), Hannover Medical School, 30625 Hannover, Germany; 4 Implant Research and Development (NIFE), Lower Saxony Centre for Biomedical Engineering, 30625 Hannover, Germany

Correction to: *Scientific Reports* 10.1038/s41598-023-46149-1, published online 09 November 2023

The original version of this Article contained an error in Figure 4, panel e, where the values of the upper x-axis were incorrect and did not match the corresponding values on the lower x-axis. The original Figure 4 and accompanying legend appear below.

**Figure 4:**
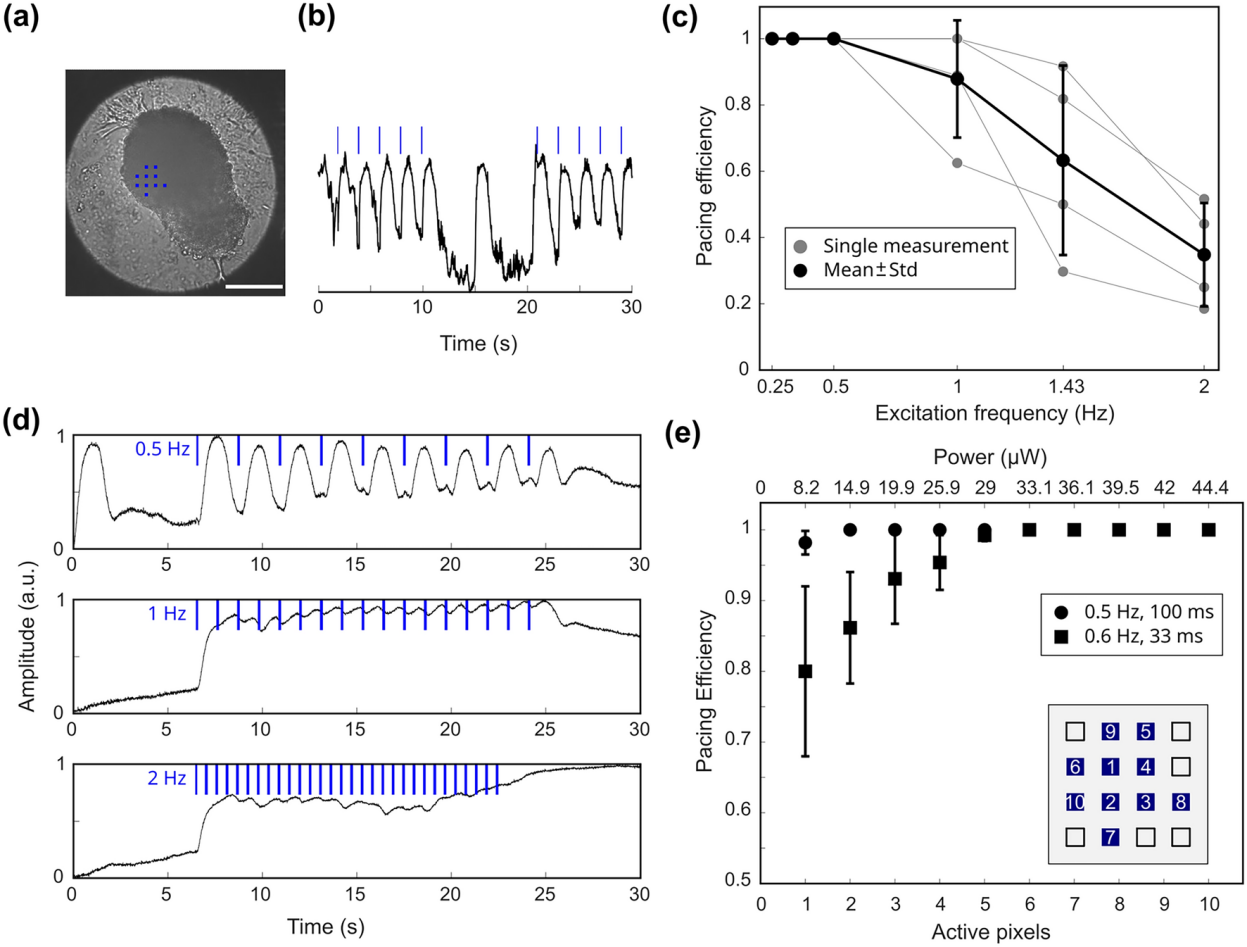
Optogenetic excitation of cardiac bodies. (**a**) Image of a cardiac body stimulated with indicated pattern of micro-LEDs (blue dots, scale bar: 200 µm) and (**b**) a contraction trace of 5 paces at 0.5 Hz with a 10 s pause followed by other 5 illuminations. Light-triggered and spontaneous peaks in contraction are clearly visible. (**c**) Dependence of pacing efficiency of cardiac bodies on pacing frequency (0.25–2 Hz, n = 4, 10 pixels). Black line represents mean values ± standard deviation. (**d**) Representative contraction traces for pacing frequencies of 0.5 Hz, 1 Hz and 2 Hz with 100 ms illumination time and 30 µW (10 pixels). Contraction becomes irregular at 2 Hz. (**e**) Pacing efficiency as a function of number of active pixels for 0.5 Hz and 100 ms illumination time (circles, n = 24) and 0.6 Hz and 33 ms illumination time (squares, n = 27). Markers represent mean values ± standard deviation. The order of switched on pixels is given in the inset.

In addition, the Project ID number was missing in the Acknowledgements section.

“This study was partly supported by the German Research Foundation, Hearing4all (EXC 2177) (Alexander Heisterkamp) and REBIRTH (EXC 62) (Ina Gruh).”

now reads:

This study was partly funded by the Deutsche Forschungsgemeinschaft (DFG, German Research Foundation) under Germany's Excellence Strategy – EXC 2177/1 – Project ID 390895286 (Alexander Heisterkamp) and REBIRTH (EXC 62) – Project ID 24102914 (Ina Gruh).

The original Article has been corrected.

