# Supplementary material for: A micro-LED array based platform for spatio-temporal optogenetic control of various cardiac models
Source: Sci Rep. 2023 Nov 9;13:19490. doi: 10.1038/s41598-023-46149-1 (PMC10636122; doi:10.1038/s41598-023-46149-1)
Supplement: Supplementary file 1 — Supplementary Figures. [file 41598_2023_46149_MOESM1_ESM.pdf]

A micro-LED array based platform for spatio-temporal optogenetic control of various cardiac models

Supplementary File

Sebastian Junge[1,4], Maria Elena Ricci Signorini[3], Masa Al Masri[1,4], Jan Gülink[2], Heiko Brüning[2], Leon Kasperek[1,4], Monika Szepes[3], Mine Bakar[3], Ina Gruh[3], Alexander Heisterkamp[1,4], Maria Leilani Torres-Mapa[1,4*]

[1]Institute of Quantum Optics, Gottfried Wilhelm Leibniz University, Hannover, 30167, Germany. [2] QubeDot GmbH, Wilhelmsgarten 3, 38100 Braunschweig, Germany, [3] Leibniz Research Laboratories for Biotechnology and Artificial Organs (LEBAO), Department of Cardiac, Thoracic-, Transplantation and Vascular Surgery, Hannover Medical School, Hannover, 30625, Germany, [4] NIFE - Niedersächsisches Zentrum für Biomedizintechnik, Implantatforschung und Entwicklung, Hannover, 30625, Germany
*torres@iqo.uni-hannover.de

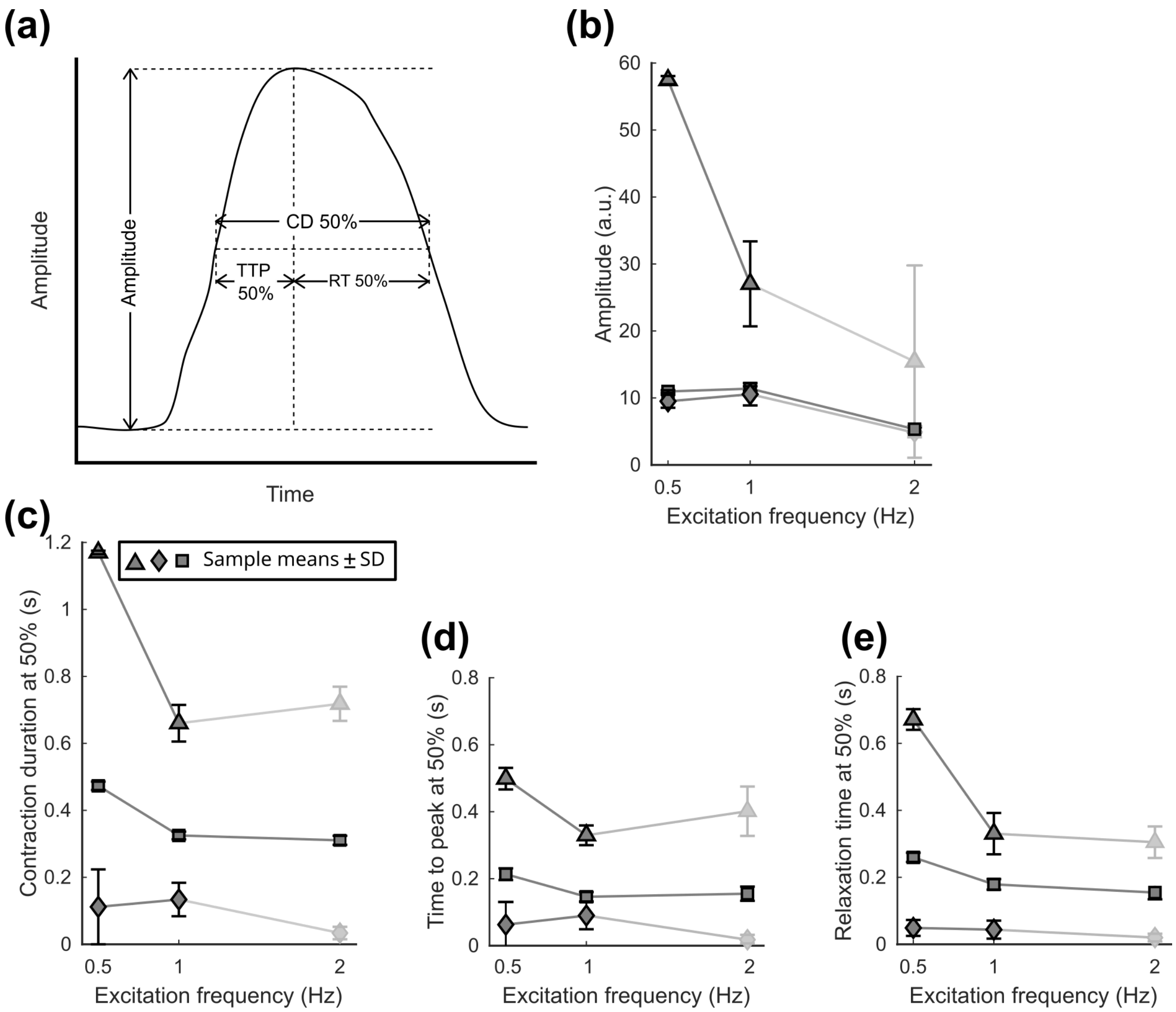

Figure S1: Contraction parameters of BCTs paced at different frequencies. The contraction amplitude (b), contraction duration at 50% (c), time to peak (d) and relaxation time (e) vary with different pacing frequencies and samples. Grey symbols indicate unsuccessful 1:1 pacing

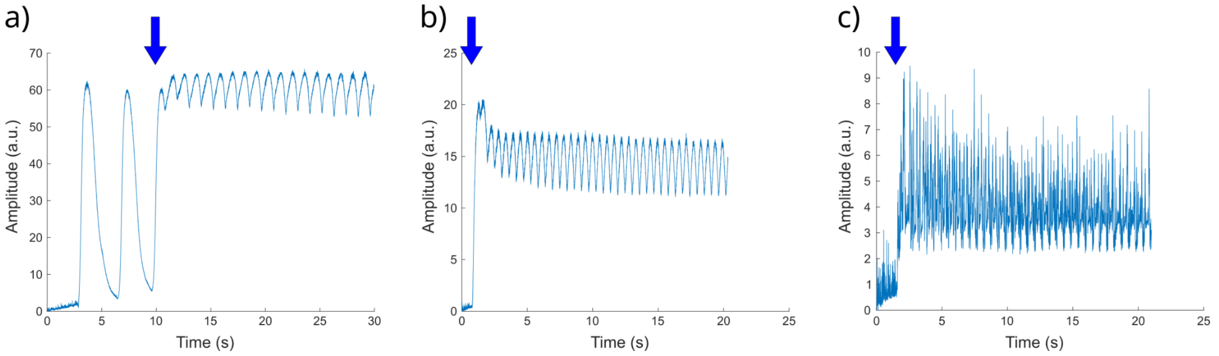

Figure S2: Pacing of the three BCT samples at 2 Hz. Micro-LEDs were switched on (at the marked time) for 100 ms every 500 ms. Though contraction is visible for all samples, one (a) does not follow the 2 Hz pacing but contracts at 1 Hz, one (b) does follow the pacing directly, and one (c) does contract irregularly with no clear distinction between contraction and relaxation.
